# Thorium and Rare Earth Monoxides and Related Phases

**DOI:** 10.3390/ma16041350

**Published:** 2023-02-05

**Authors:** Sergey V. Ushakov, Qi-Jun Hong, Dustin A. Gilbert, Alexandra Navrotsky, Axel van de Walle

**Affiliations:** 1 Center for Materials of the Universe, School of Molecular Sciences, Arizona State University, Tempe, AZ 85287, USA; 2 Joulemet Association, Pullman, WA 99163, USA; 3 School for Engineering of Transport, Energy and Matter, Arizona State University, Tempe, AZ 85287, USA; 4 Materials Science and Engineering Department, University of Tennessee, Knoxville, TN 37996, USA; 5 School of Engineering, Brown University, Providence, RI 02912, USA

**Keywords:** thorium, thorium monoxide, rare earths, rare earth monoxides

## Abstract

Thorium was a part of energy infrastructure in the 19th century due to the refractory and electronic properties of its dioxide. It will be a part of future energy infrastructure as the most abundant energy reserve based on nuclear fission. This paper discusses the solid-state chemistry of the monoxides and related rocksalt phases of thorium and the rare earths, both at atmospheric and at high pressure. The existence of solid thorium monoxide was first suggested more than 100 years ago; however, it was never obtained in bulk and has been studied mostly theoretically. Monoxides of lanthanides from Eu to Ho are ferromagnetic semiconductors sought for spintronics and were studied in thin films. La to Sm metallic monoxides were synthesized in bulk at pressures below 5 GPa. Recently, ThO formation in thin films has been reported and the stability of bulk ThO at high pressure was theoretically predicted based on first principles computations at 0 K. New ab initio computations were performed accounting for temperature effects up to 1000 K using lattice dynamics in the quasi-harmonic approximation. New computational results confirm the stabilization of pure ThO above 30 GPa and suggest the possibility of high-pressure synthesis of (Th,Nd)O at 1000 K and 5 GPa.

## 1. Introduction

The aim of this paper is to summarize the solid-state chemistry of the monoxides and related phases in thorium and rare earth systems and to present some new computational data on the stability of rocksalt (Nd,Th)O and (Y,Th)O. Thorium chemistry continues to pose critical fundamental questions, while thorium finds continued uses in refractory and nuclear technologies. The monoxides of thorium and the rare earths are of special interest because of their common (NaCl-type) structure, variable stoichiometry, and diverse electrical and magnetic properties.

Thorium was a part of energy infrastructure in the 19th century after Welsbach’s discovery of ThO_2_-based incandescent gas mantles [1]. About five billion thoria-containing mantles had been manufactured by 1930 and were in use for street lighting in London until the mid-1970s [2]. There are four times more thorium than uranium reserves, and in the 1950s, thorium was considered a strategic resource in the USA for nuclear power generation and for breeding weapons-useable uranium-233. More than 3000 metric tons of thorium nitrate were produced and stockpiled by the early 1960s, and about two tons of U-233 were separated [3]. However, U-233 was found less desirable than plutonium due to high gamma radiation from uranium-232 in its decay chain, complicating weapons fabrication and utilization [3]. Also, the new uranium deposits were discovered. The accumulated stockpile of thorium nitrate was disposed of at the Nevada Test Site [4], and separated U-233 is also aimed for disposal [3]. Hence, the systematic experimental research on the chemistry and thermodynamics of thorium has dwindled compared with uranium and plutonium. This is reflected in the number of published ternary phase diagrams: 836 for uranium, 207 for plutonium, and only 156 for thorium [5]. High-temperature heat capacities for ThC and ThC_2_ were never measured, but estimated assuming the similar shape of the *C*p curves to experimentally measured plutonium and uranium carbides [6]. Due to new regulations, the traditional use of thorium in refractory materials, alloys, optical lenses, and as filament coatings has diminished.

Nevertheless, Th chemistry keeps attracting attention, and during the last decade, ≈300 patents per year were filed that mention the use of thorium. Thorium was found to have the largest known coordination number, namely 15, in some complexes [7], and more than 70 thorium-based metal–organic frameworks have been synthesized [8]. There is also renewed interest in thorium use for nuclear energy production divorced from the build-up of nuclear arsenals. The use of domestic thorium resources was announced as India’s long-term energy strategy [9]. The commercialization of molten salt reactor (MSR) technology based on a thorium cycle is on the fast track in China, with a 2 MW experimental reactor currently being tested [10]. Thorium nitride is under development as a fuel component for compact nuclear reactors, including space reactors [11,12].

Thus, thorium will be a part of 21st century infrastructure on Earth and beyond. Thorium-based refractory materials can also find use for non-nuclear applications in space. Among metal oxides, ThO_2_ is the most stable and holds the record for the highest melting temperature (3300 °C). Many rocksalt-type thorium pnictides and chalcogenides are also excellent refractories. These attractive properties can be leveraged for the creation of ceramics and coatings tailored for operation in extreme environments in planetary probes and landers and in robotics for space exploration.

Thorium is the only actinide that has been in widespread use due to the refractory and electronic properties of its oxide. It is also by far the easiest actinide to work with, due to its low radioactivity. It is surprising to find an obsolete notion in the chemistry literature about the presence of 5*f* electrons in thorium. In the summary chapter of the 2010 edition of “The Chemistry of Actinide and Transactinide elements” [13] the editors wrote, “In the actinide series, 5*f* electrons are added successively beginning formally with thorium and ending with lawrencium. Note the qualification ‘formally.’ No compelling evidence exists to show that thorium metal, or thorium ions in solution or in any of its well-defined compounds, contain 5*f* electrons” [14].

**Figure 1:**
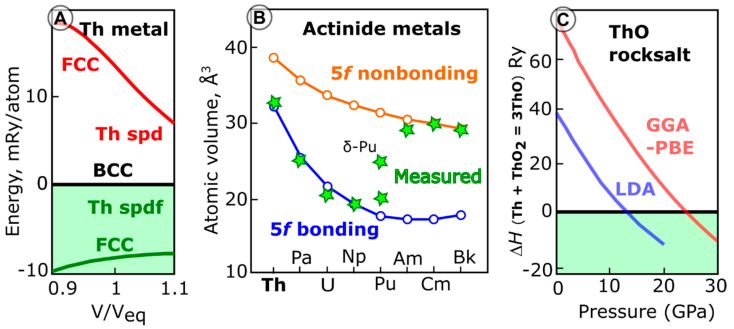
Published theoretical results on Th and ThO: (**A**) Johansson (1995) [20] computations of energy of fcc structure of thorium metal with respect to bcc, including and excluding 5*f* states occupation. (**B**) Soderlind (2014) [21] computations for actinides’ atomic volume in metals vs. experimental values, indicating a change from itinerant to localized nature of 5*f* electrons. (**C**) Sun’s (2015) [22] prediction of enthalpy of ThO stabilization between 14 and 22 GPa.

Physicists may disagree, at least with respect to thorium metal. Indeed, the first measurements of magnetization of a Th single crystal [15] (de Haas van Alphen effect), performed in the 1960s, were interpreted [16] without including 5*f* states in Th. This led to the notion that Th is more aligned with 4*d* transition metals like Zr. However, later measurements of reflectivity [17] and X-ray photoemission [18] could not be satisfactorily modeled without 5*f* occupation. In the 21st century, electron energy loss spectroscopy measurements provided the range of 0.6 to 1.3 5*f* electrons in thorium metal, depending on the background removal procedure [19]. Several theoretical studies considering 5*f* states in thorium metal have followed the case. One of the most notable is a 1995 paper by Johansson [20], showing that the ground state electronic structure of metallic face-centered-cubic (fcc) thorium can only be reconciled when 5*f* orbitals are included in calculations (Figure 1A). Thus, we can argue that the disqualification “formally” in the statement quoted above should be removed, and thorium, de facto, deserves its place as the first element with 5*f* state occupation. This brings thorium back into the fundamental “actinide challenge”: electron distributions between 5*f*, 6*d,* and 7*s* shells, their localized vs. itinerant behavior, and their involvement in bonding. 

The existence of thorium and rare earth monoxides in the vapor phase at high temperatures is well established [23,24]. Recently, gaseous ThO attracted physicists’ attention in connection with Th electron electric dipole moment determination [25]. The thermodynamic properties of gaseous lanthanide and actinide monoxides were studied extensively and recently summarized by Konings et al. [26]. The first report suggesting the synthesis of solid thorium monoxide dates to 1908 [27]; however, it is still considered a “long-sought” phase [28] with unknown properties. The properties of solid thorium monoxide are of interest not only as insight into bonding in actinides, but for the thermodynamic modeling of oxygen solubility in rocksalt-type ThC and ThN, studied as advanced nuclear fuels [29,30,31,32].

The chemistry of rare earth (RE) elements (lanthanides, yttrium, and scandium) is often used as a guide for actinides. Among RE, only europium is known to form bulk solid monoxide at ambient pressure. EuO has a NaCl-type (rocksalt) structure, it can be synthesized in bulk at ambient pressure and grown as single crystals, and its thermodynamic, magnetic, and electronic properties are well established [33]. 

Rocksalt-type monoxides of La, Ce, Pr, Nd, Sm, and Yb were recovered in bulk from high-pressure synthesis [34] and the magnetic properties of PrO, NdO, and SmO were measured [35]. However, the high-pressure synthesis and magnetic measurements were performed more than 40 years ago and never repeated. In the last decade, most rare earth monoxides have been prepared at low pressures as epitaxial thin films [36,37,38,39,40,41,42,43,44]. Superconducivity was detected in LaO [42]; Gd, Tb, and Ho monoxides were found to be ferromagnetic semiconductors with high Curie temperatures [36,37,38]—materials highly sought for applications in spintronics [45,46].

Despite the metastability of bulk ThO and most other REO at ambient pressure, their thermodynamic description is required in the CalPhaD (Calculation of Phase Diagram) approach to calculate binary, ternary, and higher order phase diagrams [47]. In the present work, we review relevant systems with Th and RE and prior studies on thorium and rare earth monoxides. An emphasis is placed on experimental data; however, recent ab initio predictions of the high-pressure stability of YO [48] and ThO [22] are discussed and new computations indicating the stability of bulk (Th,RE)O at pressures as low as 5 GPa are presented.

## 2. Th-RE Systems

Thorium melts at 1750 °C. This is the highest melting point among actinide and lanthanide metals [49]. At room temperature, Th is stable in an fcc structure up to 100 GPa [50]. At ambient pressure, the Th ground-state fcc structure transforms into a body-centered cubic (bcc) structure at 1360 °C.

The volume change in the high-temperature fcc–bcc transformation was found to be zero within the accuracy of the measurements, and the pressure—temperature boundary for fcc–bcc transformation and pressure dependence of Th melting temperature have not been determined [51]. Among actinides and lanthanides, only thorium, actinium, einsteinium, and cerium have an fcc structure at ambient pressure and room temperature; La transforms to fcc at high temperature [49] (Pr, Nd, and Sm transform to fcc at high pressure and temperature [51]). All studied actinides and lanthanides from La to Dy transform to bcc structure before melting [19,49,51,52]. 

Th-RE binaries are characterized by extensive solid solutions in the Th fcc structure and continuous solid solutions in the high-temperature bcc structure. The notable exceptions are Yb and Eu, which, essentially, are not miscible with Th in solid or liquid state [53]. This anomaly was experimentally established by Badaeva et al. in 1969 [53] and was not investigated further either experimentally or theoretically. She interpreted this as the consequence of the half-filled and fully filled 4*f* orbitals of Eu and Yb. This is specific to metallic bonding, as apparent from the oxide phase diagrams discussed below. 

## 3. Th-RE-O Systems

**Figure 2:**
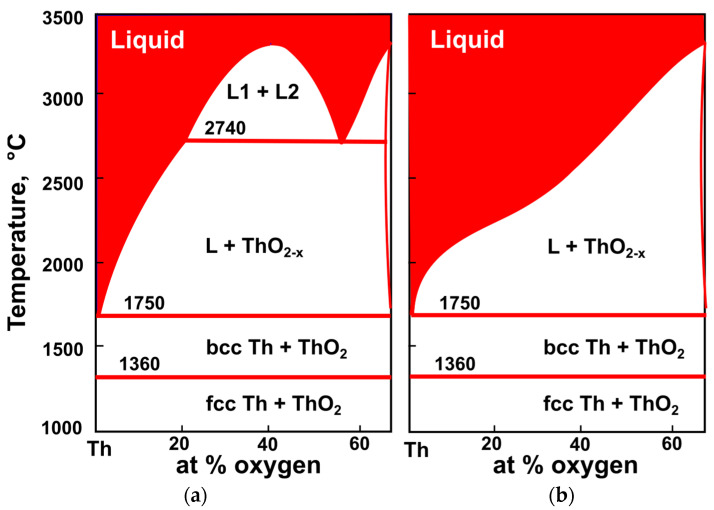
Thermodynamic assessments of Th-O system (**a**) after McMurray et al. [55]; (**b**) after Bergeron et al. [56].

Th-O is the only system with thorium for which CalPhaD thermodynamic assessments are available (Figure 2). Notably, there are two distinct assessments which mostly rely on the same dataset from experiments performed by Benz at Los Alamos in the 1960s [54]. The first assessment was published by a group from Oak Ridge and Los Alamos as part of the U-Th-O systems assessment [55]. 

They followed the tentative immiscibility gap suggested in Benz’s study. The second assessment by Bergeron et al. [56] was part of the description of the Th-U-Pu-O system and included several new measurements. The new data were not conclusive about the presence of a monotectic, and the authors modeled the system without a miscibility gap but stated that new experiments would be desirable. Most of the studies on Th phase diagrams were performed in the 1960s and early 1970s, with phase equilibria determined by microscopic examination of quenched samples. In Benz’s experiments [54], the monotectic reaction at 2740 °C was tentatively suggested from observed the microstructure (Figure 2) and by analogy with a U-O phase diagram. The major difficulty in interpretation was also due to the reaction with ThO_2_ crucibles.

ThO_2_-RE_2_O_3_ systems are characterized by large fields of defect fluorite (Th,RE)O_2−x_ solid solutions, reaching ≈ 50 mol% RE_2_O_3_ at eutectic temperatures (typically around 2400 °C). Continuous fluorite-type solid solutions are observed for the ThO_2_-CeO_2_ system. Notably, no anomalous immiscibility is observed in the studied ThO_2_-Yb_2_O_3_ phase diagram [57].

## 4. Thorium and Rare Earth Rocksalt Phases

Rocksalt structure, also known as B1 or NaCl-type structure, can be described as two interpenetrating fcc sublattices of metal and non-metal atoms. Reported thorium rocksalt phases include ThO, ThC, ThN, ThS, ThAs, ThP, ThSb, and ThSe [58,59,60]. One could mistakenly conclude that such variety of rocksalt phases in thorium compounds is due to the fcc structure of thorium metal, but in fact, all other studied actinide monopnictides and monochalcogenides also form rocksalt phases, as well as rare earth monoxides, nitrides, and Ti, Zr, and Hf nitrides and carbides [61]. Most of these compounds have metallic conductivity but do not inherit ductility. Although they often have a range of possible stoichiometries, they are not interstitial Hagg phases [60], since most of these metals are not fcc and do experience changes in their positions to form the fcc sublattice in a rocksalt phase. 

These compounds, in which metal electrons are drained from metallic bonding to form ionic/covalent bonds with non-metals, are fascinating from both fundamental and applied viewpoints. For thorium-rich phases, the changes in the role of 5*f* electrons with changes in the degree of metallic—ionic—covalent bonding is the fundamental aspect. The fact that these rocksalt phases retain metal-like thermal and electrical conductivity but gain remarkable increase in melting temperature and bulk modulus make them useful for a variety of applications from nuclear fuels to ultra-high temperature ceramics.

RE-N and Th-RE-N systems provide examples of continuous solid solutions between Th and RE nitrides in a rocksalt phase. Holleck [62,63] reported solid solutions in ThN-REN systems with Y and La-Nd. These findings suggest a possibility of complete miscibility in (Th,RE)O solid solutions. Extensive solubility is corroborated by continuous solid solutions between Th and RE metals in high-temperature bcc structure, with the exception of Yb and Eu, noted above. 

### Rocksalt Solid Solutions in Th-C-N-O Systems

Thorium monocarbide and thorium mononitride crystallize in NaCl-type rocksalt structure and form a complete solid solution. ThC is stable in rocksalt structure to ≈58 GPa [64]. The limits of oxygen solubility in thorium carbide at ambient pressure are unknown. Henney et al. [65] reported the synthesis of thorium carbide with carbon deficiency, ThC_0.7_ to ThC, with corresponding lattice parameters increasing from 5.295 Å to 5.344 Å, and stated that “circumstantial evidence indicates that oxygen solubility is responsible for the lower cell sizes.” ThN cell parameter is 5.160(2) Å, as measured for a well characterized carbon and oxygen free sample [66]. However, values from 5.159 to 5.196 Å have been reported in the literature [67]. The higher cell parameters are likely due to carbon impurity, since the nitridation of ThC is a common method to synthesize ThN [68]. The solubility of oxygen in ThN is unknown, but considered to be less than in ThC, at least at ambient pressure. 

**Figure 3:**
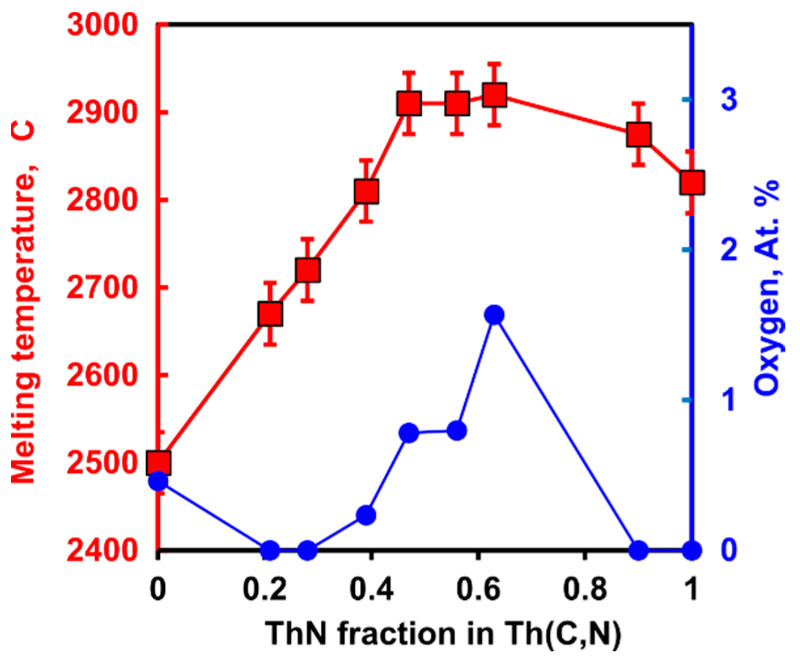
Melting temperature of Th(C,N) solid solutions and oxygen content in analyzed melted products after Benz (1968) [75].

The Th-C binary was studied by several groups [69,70,71]. Th-N, Th-C-N, and Th-N-O systems were studied only by Benz [72,73,74] in Los Alamos. The congruently melting thorium carbide stoichiometry was reported to be ThC_0.97_, with a melting point of 2500 ± 35 °C. Below 1000 °C, ThN is essentially a line compound with negligible solubility of oxygen. ThN melts congruently at 2820 ± 30 °C at 2 Bar N_2_, and at 2810 ± 30 °C at 1 Bar. Benz [75] reported melting point maxima for rocksalt ThC-ThN solid solution in ThC_0.35_N_0.65_ composition with a melting temperature of 2910 ± 35 °C (Figure 3). It indicates a strong deviation from ideal solution in solid, liquid, or both. Interestingly, the increase in melting temperature correlates with the higher oxygen content reported in the analysis of melted samples (Figure 3). 

## 5. Thorium Monoxide

The background on solid actinide monoxides is summarized succinctly by Petit et al. in a 2010 *Physical Review* publication: “There exists to date no convincing evidence that actinide oxides can form in the 1:1 stoichiometry” [76]. Petit et al. cited earlier reviews and some earlier reports on the synthesis of uranium monoxide, which were later proven to be uranium oxycarbide, oxynitride, or oxycarbonitride. They did not cite, however, experimental work on ThO and UO published by Ackerman and Rauh [77] from the Argonne National Laboratory. New experimental evidence of ThO formation in thin films at near-ambient conditions has been recently published [28], and bulk thorium monoxide was predicted to be stable in the NaCl structure at high pressure (Figure 1C) [22].

The stability of ThO, computed by density functional theory (DFT) methods, is attributed to the transfer of thorium’s 7*s* and 6*d* electrons to 5*f* orbitals and 6*d* -2*p* hybridization [22]. The computational prediction of the pressure-driven stabilization of PuO promptly followed [78]. These results challenge the current understanding of actinide chemistry, since, until this prediction, “the only hope of synthesis of actinide monoxides would appear to be the high-pressure route for AmO and CfO, an extremely demanding synthetic procedure” [6]. 

The experimental reports on ThO are summarized in Table 1, together with reference values for cell parameters for metallic Th and ThO_2_ phases. In most cases, ThO was reported to be prepared either on the decomposition of amorphous solids produced by the reaction of metallic Th with hydrochloric acid, or obtained on the surface of metallic Th. 

The only exception is the report from 1954 by Hoch and Johnston [81] on the formation of ThO above 1700 °C on the surface of thoriated tungsten cathodes. Based on high-temperature diffraction measurements, Hoch and Johnston reported ThO formation from liquid Th and ThO_2_ at high temperatures and its decomposition back to metal and dioxide on cooling. The possibility of ThO stability at high temperature was ruled out by Benz experiments in 1960s [54] on Th-O phase equilibria (see Section 3 for details). The unit cell parameter for ThO reported by Hoch and Johnston [81] (4.31 Å) is much lower than in other reports (5.24–5.41 Å), and it is also lower than the room temperature lattice parameter for pure metallic Th in fcc structure. However, this lattice parameter is close to the value for the high-temperature Th bcc phase (4.11 Å) (Figure 2), not yet known at the time of the Hoch and Johnson [81] study. There are no experimental data on the Th-O system or the Th + ThO_2_ → 2ThO reaction at high pressure. The published work on ThO from low-temperature synthesis and growth as films on Th is discussed in detail in two separate sections below.

### 5.1. Residues from Th Dissolution in HCl

Metallic Th does not dissolve completely in hydrochloric acid, leaving a black residue. The hypothesis that this residue is composed of lower oxides of thorium was put forward more than 100 years ago by Werner von Bolton [27]. This incomplete dissolution of metallic thorium in HCl was rediscovered during its manufacturing for irradiation and U-233 separation [59]. The initial notion was that the residue is simply metal particles coated with ThO_2_ [84]. The study, performed at the Argonne National Laboratory by Katzin in 1958 [82], supported von Bolton’s hypothesis and concluded that the residue is basically ThO. Katzin reported that the ThO structure was clearly identified by neutron diffraction as ZnS type, with cell parameters similar to ThO_2_ (Table 1); however, the details of neutron diffraction measurements were not reported. In the same year, a study of the residue on Th dissolution in HCl was published by Karabash [85]. He reported that the black residue is thorium hydroxyhydride ThH(OH)_2_, which oxidizes to ThO_2_ on heating in air to ≈150 °C but appears stable in a vacuum. Four years later, another study on the residue was performed by Newbury and Searcy [86], defining its composition as ThO_1.3_Cl_0.7_H_1.3_.

The latest investigation on the decomposition of black Th residue was also performed in the Argonne National Laboratory, 15 years after Katzin’s experiments. Ackermann and Rauh [77] dissolved ≈1 g of metallic Th in concentrated HCl, obtained a black high surface area precipitate, and characterized its decomposition by X-ray diffraction (XRD) and thermogravimetry. They produced an empirical formula for the residue, ThO∙HCl∙H_2_O, and found that upon heating to 1200 °C in a vacuum, it decomposes to a mixture of rocksalt ThO, metallic Th, and fluorite ThO_2_ phases, with ThO disappearing at higher temperatures. Ackermann and Rauh [77] published an XRD pattern and reported the lattice constant for ThO to be 5.302 Å.

### 5.2. Rocksalt ThO Films on Metallic Th Surface

The first evidence on rocksalt ThO films was obtained at Ames during the Manhattan project and was included in Rundle’s 1947 paper [60]. Rundle refers to thorium monoxide in the scope of a new interpretation of structure—property relations in interstitial metallic carbides, nitrides, and oxides having the sodium chloride structure. Rundle does not explicitly report a lattice parameter for ThO, but does publish metal–metal distances which correspond to a lattice parameter 5.24 Å for rocksalt ThO, compared with 5.07 Å for fcc thorium metal, which is close to the currently accepted value (5.084 Å [83]). Rundle did not indicate how rocksalt ThO was obtained, but in reference to his work, Katzin [82] stated that it was “formed on the surface of freshly cleaned thorium metal”. Seventy years after Rundle’s [60] report, the growth of ThO thin films was reported by the Los Alamos Laboratory. He et al. [28] performed experiments on the controlled oxidation of metallic thorium with in situ neutron reflectometry measurements emerging from the study of oxidation of metallic Th using neutron reflectometry. ThO was formed after one hour of exposure of metallic Th to a ≈100 ppm O_2_ in an Ar mixture at 150 °C. From the modeling of the changes in experimentally measured neutron scattering length density (SLD) distribution, the composition can be derived as ThO_0.75_ with the cell parameter ≈5.41 Å. When the thickness of the oxide layer exceeded ≈100 nm, thorium dioxide was observed [28].

## 6. Rare Earth Monoxides

The monoxides of La, Ce, Nd, Sm, Eu, Y, and Yb with rocksalt structures were reported in the 1950s and 1960s [87,88]. They were synthesized by the reduction of sesquioxides with carbon or corresponding rare earth metals. However, it was later established that only divalent EuO can be synthesized at ambient pressure, and reported monoxides of other rare earth elements were, in fact, oxycarbides or oxynitrides [89].

Rocksalt EuO is the only rare earth monoxide known to be thermodynamically stable at ambient pressure (i.e., it will not decompose to Eu and Eu_2_O_3_, although it will oxidize in the presence of oxygen and, as all rare earth oxides, will react with water vapor, forming hydroxides). EuO has been synthesized by direct combustion of the metal, reaction of Eu with Eu_2_O_3_, and grown as single crystals with varying stoichiometry from Eu-Eu_2_O_3_ melt [90,91]. Close to stoichiometric EuO is a semiconductor, but EuO_0.7_ shows metallic conductivity. Melting temperatures of up to ≈2000 °C have been reported for oxygen-rich compositions. EuO is the only rare earth monoxide for which formation enthalpy [92,93] and heat capacity [94] have been measured [26].

### 6.1. Rocksalt REO from High-Pressure Synthesis

YbO and lanthanide monoxides from La to Sm were synthesized at high pressure by Leger et al. [34]. La, Pr, Nd, and Sm monoxides were obtained at 4–5 GPa from stoichiometric mixtures of rare earth metals and sesquioxides in a compressed gasket (“belt-type”) apparatus. The reaction temperatures (800–1000 °C) were chosen to not exceed melting temperatures of corresponding lanthanide metals. The synthesis of pure YbO has been accomplished at pressures as low as 1 GPa [95]. Cerium monoxide was synthesized from metallic Ce and CeO_2_ at 1.5 GPa and 750 °C. CeO was only prepared in a mixture with Ce_2_O_3_ and unreacted metallic Ce; the other synthesized REO were reported to be single phases [96]. REO were not reported to decompose into metal and sesquioxide at ambient pressure [34]. Instead, hydroxide formation was observed, increasing from Sm to La, which is similar to the trend in rare earth sesquioxide affinity to reactions with water vapor [97]. The high-pressure syntheses were carried out in BN crucibles and reaction products were analyzed for H, C, and N to assure that no hydrides or carbonitrides were formed.

Yb and Eu are formally divalent in monoxides, have brown and red colors, and are semiconductors at ambient pressure; the rest of the synthesized REO have a metallic golden luster and the authors argued that the rare earth is trivalent [34]. The supposition of trivalent cations in the monoxide may seem bizarre, since we are used to the bonding in oxides being mostly ionic. In synthesized La-Sm monoxides, however, while overall formal oxidation state is +2, the RE is considered to be in trivalent state, with the third electron delocalized in metallic bonding; these compounds can be described as RE^3+^(O^2−^)(e^−^), according to Morss and Konings [33].

An ambiguity remained about SmO since Sm is divalent in the rocksalt monosulfide. SmS is a semiconductor but is known for its fascinating pressure-driven “black-golden” transition which can be induced by mere polishing [98]. It appears to be caused by Sm going into the trivalent state, switching SmS from semiconductor to metal, while still keeping the rocksalt structure [98]. Since golden SmO has metallic conductivity, it was surmised that Sm is mostly trivalent in the monoxide, which was confirmed by X-ray absorption spectroscopy [35]. Leger et al. [34] also attempted the synthesis of Gd, Dy, and Tm monoxides at 1 to 8 GPa and 600–1200 °C, but did not succeed. 

Rocksalt CeO was further studied in a diamond anvil cell and found to be stable at least up to 25 GPa, which was the maximum pressure used in the experiments [99]. Rocksalt NdO was also obtained in shock compression experiments [100,101,102]. Comparing lattice parameters of NdO synthesized in shock compression with those under static 5 GPa loads (Table 2), the authors suggested that in NdO retrieved from explosions, the neodymium is partially divalent [101]. We did not locate any other reports on high-pressure synthesis, thermodynamics, and property characterization of bulk rare earth monoxides.

In a review on the thermochemistry of binary rare earth oxides, Morss and Konings stated that solution calorimetry experiments were performed on several NdO samples prepared by Leger’s group, “however their results were unusually exothermic, indicating that metallic Nd was present, and the non-reproducible measurements indicated that the samples were not sufficiently homogeneous to warrant further study” [33].

### 6.2. Rocksalt REO Films from Pulsed Laser Deposition

Experiments on the high-pressure synthesis of rare earth monoxides described above were performed more than 40 years ago and were never repeated. However, in the last decade, many rare earth monoxides were synthesized as epitaxial thin films at low pressures, motivated by their potential use in spintronics. Most of the experiments were performed by a group in Tohoku University using pulsed laser deposition (PLD) [38,40,41,42,43,44]. The films were prepared in an ultra-high vacuum on a variety of substrates including CaF_2_, LaAlO_3_, and YAlO_3_, typically held at temperatures 200–400 °C. The oxygen pressure varied from less than 10^−9^ to 10^−7^ Torr, depending on whether metallic or oxide targets were used for evaporation. The thickness of synthesized REO films varied from 6 nm for CeO to 200 nm for YO (Table 2).

The discovery of superconductivity in LaO films [42] and ferromagnetism in semiconducting GdO films [38] prompted a number of experiments on the growth of films on different substrates and background oxygen pressures to evaluate their effects on critical superconductivity and Curie temperatures. Thin films were grown both in compressive and tensile epitaxial strain, which may indicate that epitaxial growth is not critical for the low-pressure synthesis of REO, and they can potentially be synthesized as separate grains or nanoparticles. In oxygen deficient rocksalt REO_1−x_, the cell parameters were found to increase with decreasing oxygen content. 

**Figure 4:**
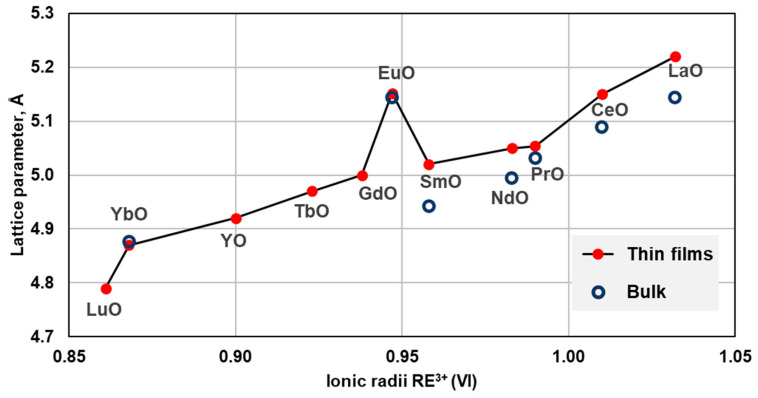
Cell parameters of rocksalt-type REO from high-pressure synthesis and thin films. The symbol sizes exceed reported uncertainties. Reported cell parameter for HoO film (a = 5.04 Å) is not included in the plot as a possible outlier. The references are listed in Table 2.

In Figure 4, lattice cell parameters of rocksalt REO grown as thin films are compared with those for bulk monoxides from high-pressure synthesis and with EuO, prepared at ambient pressure. For monoxides from lanthanum to samarium, for which data both from thin films and bulk are available, cell parameters from the thin films are larger than from the bulk. La to Sm monoxides were reported to be metallic both in bulk and in thin films. 

YO and heavy lanthanide monoxides from Gd to Lu were synthesized only in thin films. They were found to be semiconductors, similar to EuO and YbO, which are semiconductors in bulk and in thin films. From X-ray photoelectron spectroscopy (XPS) measurements, the authors concluded that rare earths from Gd to Lu are in the divalent state in monoxides [44,107]. This is in stark contrast with metallic monoxides from La to Sm, in which the rare earths are considered to be trivalent. The excellent agreement between lattice constants for Yb^2+^ and Eu^2+^ monoxides in bulk and in thin films prompts the question whether larger lattice constants of REO from La to Sm in thin films vs. bulk indicate mixed valence states in these REO as well.

## 7. Ferromagnetism and Superconductivity in Thorium and Rare Earth Monoxides

Magnetic measurements on actinides and rare earths monopnictides and monochalcogenides were reviewed by Vogt and Mattenberger [108]. Since these compounds share the rocksalt-type structure, solid solutions with nonmagnetic elements (e.g., Th, La, Lu, and Y) can be used to investigate the effects of dilution and interatomic distances on magnetic properties [109,110]. Single crystals are used for most informative measurements, since even in cubic structure as NaCl, magnetic anisotropy is often observed. 

In 1961, rocksalt EuO was discovered to be ferromagnetic below 77 K with a magnetic moment comparable to iron, gadolinium, and dysprosium [111]. This prompted investigations into the magnetic properties of other rocksalt-type Eu chalcogenides, which were also found to exhibit ferromagnetic behavior, albeit at lower temperatures than EuO (Table 3). Growing EuO crystals with additions of La, Gd, Ho, or Y increases their Curie temperature (T_c_) to a maximum of 135 K [112]. La-doped EuO crystals were found to incorporate less than 0.5 mol% LaO, but demonstrated the same increase in T_c_ as EuO crystals containing 3.4 and 5 mol% Gd and Ho, respectively [112].

50 years later, in a quest to lower currents in integrated circuits, ferromagnetic semiconductors became highly sought after materials in a new field of spin-transport electronics (spintronics) [45]. In 2010, Miyazaki et al. [105] reported epitaxial La_0.1_Eu_0.9_O as a ferromagnetic semiconductor with a T_c_ of 200 K; this was the highest Curie temperature reported for these materials at the time. Since Eu is divalent in monoxide, La^3+^ introduction contracted the lattice parameter from 5.152 to 5.116 Å, as was intended to enhance the hybridization of Eu 4*f* states. Authors noted that the increase in T_c_ with doping cannot be quantitatively explained [105]. In the last decade, new ferromagnetic semiconductors were discovered in Gd, Tb, and Ho monoxides; weak ferromagnetism was also detected in thin films of metallic PrO and NdO [40,104]. 

The Curie temperature for GdO prepared in thin films [38] was found to be higher than for any other rocksalt monochalcogenide and monopnictide and slightly lower than for metallic hcp Gd (Table 3). Magnetic ordering was not detected in the first measurements on polycrystalline samples of Pr and Nd monoxides from high-pressure synthesis [35]. La, Ce, Pr, Nd, and Sm monoxides were synthesized in bulk by Leger [34] and were reported to be metallic. Thin films of these REO were found to be metallic as well [43]. However, monoxides of Gd, Tb, Ho, Y, Yb, and Lu, synthesized only in thin films, were reported to be semiconducting [37]. Similarly to EuO, electric conductivity was greatly enhanced in oxygen deficient samples [44].

Surprisingly, the Curie temperatures of many of the ferromagnetic RE monoxides align closely with the critical temperatures (T_C_ or Néel temperatures, T_N_) of parent metals (Table 3), typically within ≈10%. Magnetism in RE metals has complex origins, arising from a competition between the oscillatory Ruderman–Kittel–Kasuya–Yosida (RKKY) exchange interaction, the magnetocrystalline anisotropy, and magnetoelastic effects. These factors result in a temperature-dependent magnet landscape that includes ferromagnetic phases as well as non-colinear ferro- and antiferromagnetic helical phases and fan phases [116]. The RKKY coupling in RE metals occurs due to an effective exchange interaction between the localized 4*f*-electrons mediated by the delocalized 5*d* and 6s conduction electrons. The common metallic character of the RE monoxides allows a similar interaction to exist and may explain the similarity of their critical temperatures. A more comprehensive consideration of the REO Fermi surface would be necessary to confirm the underlying magnetic interactions.

No experimental measurements of the magnetic or superconducting properties of ThO in thin films or nanoparticles were reported. Ab initio computations indicate that ThO is metallic with the presence of Th 5*f* states in the occupied bands [22,117,118]. Thorium metal is a classic type-I superconductor which exhibits a complete Meissner effect below 1.4 K and critical magnetic field 0.15 T. It was first measured by Meissner himself [119]. The metal is paramagnetic with nearly temperature-independent magnetic susceptibility. Among superconducting elements, Th is the only one which forms significant solid solutions with magnetic 3*d*, 4*f*, and 5*f* elements, and was used to study the depression of superconductivity in solid solutions with Ce, Pr, Gd, Tb, and Er [109]. Pressure suppresses superconductivity in Th to below 1 K at 8 GPa [120]. Rocksalt thorium carbonitrides also were reported to exhibit a small temperature-independent paramagnetism [121] based on measurements above 80 K. ThN is superconducting below 3.2 K, but no superconductivity was detected in ThC with measurements down to 1.2 K [122]. The superconductivity critical temperature was found to increase in Th(C,N) solid solutions to a maximum of 5.8 K in ThC_0.78_N_0.22_ [123].

LaO was the first experimentally reported rare earth binary oxide superconductor [42]. Type-II superconductivity was discovered in rocksalt LaO at ambient pressure below 5 K. This was measured by Kaminaga et al. in 2018 [42] on epitaxial thin film grown by pulsed laser deposition and capped in situ with an aluminum oxide layer to prevent oxidation. Bulk LaO was synthesized earlier under high pressure; however, no measurements of the magnetic field response, corresponding to ferromagnetism or superconducting field rejection, were reported, probably due to the sample instability in air. The superconducting temperature in LaO is higher than in any other rocksalt La monochalcogenides [42], but 1 K lower than in metallic La [124]. Superconductivity in rocksalt LaO decreases under compressive strain [42], indicating that hydrostatic pressure would decrease superconductivity as well, in contrast to double-hexagonal-closed-packed (dhcp) La, but analogous to superconductivity in rocksalt TiO [125] and fcc Th. Superconductivity, with critical temperature decreasing with pressure, was also predicted for rocksalt yttrium monoxide [48]; however, it was not observed in YO thin film, which was reported to be a narrow-gap semiconductor [44]. 

Among rare earth elements, only La is known to be superconductive at ambient pressure, with a critical temperature of 6 K in its dhcp structure [124]. In contrast to fcc Th, pressure promotes superconductivity in dhcp La (13 K at 15 GPa) [126]. Superconductivity was also reported in cubic decahydrates of lanthanum and thorium at ≈170 GPa (250 K for LaH_10_ vs. 161 K for ThH_10_) [127,128]. Y, Sc, Lu, and Ce become superconducting under pressure [126,129,130]. Metallic Sc and Y hold the record for the highest superconductivity among elements (≈20 K at ≈100 GPa) [131]. 

## 8. New Computational Results

ThO was predicted to be thermodynamically stable above 12–22 GPa with respect to Th and ThO_2_ [22]. This is based on density functional theory (DFT) [132] computations performed at 0 K. Here, we report new computational results of the free energy of ThO and (Th,RE)O formation at pressures and temperatures achievable in a multianvil apparatus.

While obtaining energy at 0 K requires only static DFT calculation, introducing temperature dependency requires sampling multiple configurations and the calculation of reaction-free energy for all reactants and products. The computations were automated with Alloy Theoretic Automated Toolkit (ATAT) code [133], which interfaces with the Vienna Ab-initio Simulation Package (VASP) [134]. We employed the PBE [135] functionals, which in the 0 K computations by Sun et al. [22], resulted in a higher value of stabilization pressure compared with LDA. The pseudopotentials used are [Xe5d^10^4f^14^]6s^2^6p^6^6d^2^7s^2^ for Th, [Ar3d^10^]4s^2^4p^6^4d^1^5s^2^ for Y, and [Kr4d^10^]5s^2^5p^6^4f^4^6s^2^ for Nd, with a planewave energy cutoff of 500 eV. The effect of temperature was evaluated using lattice dynamics calculations in the quasi-harmonic approximation [136], known to provide reliable results at temperatures below ≈1000 K. Gibbs free energies (ΔG) of the reactions were calculated with ATAT by including the vibrational contribution from lattice dynamic calculations based on the ATAT *fitfc* utility [137]. Additional details on ATAT tools can be found in a user guide [138].

We calculated ΔG for the reactions of fcc Th metal with fluorite-type ThO_2_ resulting in the formation of rocksalt-type (*Fm*-3*m*) ThO, and with Nd_2_O_3_ and Y_2_O_3_ resulting in rocksalt (Th,RE)O solid solutions. The initial structures for Nd and Y sesquioxides were chosen to be *P*-3*m*1 (A-type) and *I*a-3 (C-type, bixbyite), respectively, in accordance with the accepted stable structures for these compounds.

**Figure 5:**
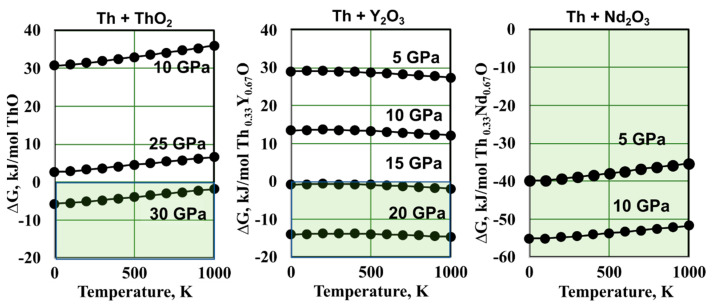
New computational results: free energy for reactions of ThO and (Th,RE)O formation from Th metal, ThO_2_, Y_2_O_3_, and Nd_2_O_3_. The fields of thermodynamic stability of the rocksalt solid solutions are marked in green.

Our results for ThO confirmed its high-pressure stabilization and indicated a shallow positive P–T slope for the reactions of ThO and Th_0.33_Nd_0.67_O formation (Figure 5). The reaction for Th_0.33_Y_0.67_O formation has a shallow negative P–T. The slightly higher pressure value for ThO stabilization at 0 K in our calculations compared with previously published PBE results (Figure 1C) can be attributed to our use of 12 electron pseudopotential for Th vs. the full potential computations performed by Sun et al. [22]. The predicted ThO lattice parameter was 3.5% larger than for fcc Th metal, compared with 5% increase in ThC formation and 1.5% increase in ThN. The small (less than 10%) difference in the calculated rocksalt ThO phase with cell parameters of experimentally synthesized REO supports the hypothesis that a continuous (Th,RE)O solid solution can be formed. Our results indicate that while the synthesis of pure ThO can only be achieved above 30 GPa, Th + Y_2_O_3_ become stable in a rocksalt-type solid solution above 15 GPa, and Th + Nd_2_O_3_ should produce a rocksalt-type solid solution at 5 GPa with free energy of the reaction −35 kJ/mol Th_0.33_Nd_0.67_O at 1000 K.

## 9. Summary and Future Directions

Thorium is by far the easiest actinide to work with; however, the properties of solid thorium monoxide are still unknown. The existing experimental data indicate that ThO can be synthesized in rocksalt structure in thin films at ambient pressure upon the controlled oxidation of metallic thorium and occur as a minor phase upon the decomposition of thorium oxychlorides formed on the dissolution of Th in HCl. The new computational results confirm the prediction of rocksalt ThO stabilization at pressures above 30 GPa and indicate negative free energy in the formation of the rocksalt (Th,RE)O solid solutions by reaction of metallic Th with Nd_2_O_3_ at 5 GPa, and with Y_2_O_3_ at 15 GPa. Experimental thermodynamic data on ThO and its solid solutions in thorium and rare earth carbonitrides are required for benchmarking ab initio computations and the modeling of new Th-containing fuels and refractories. 

Among solid rare earth monoxides, only Eu forms divalent monoxide in bulk at ambient pressure. Thermodynamic, magnetic, and electronic properties of EuO are well established. Monoxides of La, Ce, Pr, Sm, and Yb were synthesized in thin films at low pressures and in bulk at pressures of 5 GPa or lower and found to be metallic. Monoxides of Gd, Tb, Ho, Y, Yb, and Lu were synthesized only as thin films and found to be narrow gap semiconductors. PrO and NdO in thin films exhibit itinerant ferromagnetism below 28 and 19 K, respectively, which was not detected during the study of the bulk samples from high pressure. LaO was found to be superconducting in thin films; no studies on bulk sample from high-pressure synthesis were reported. YO was predicted to be stable in rocksalt phase at a pressure higher than 10 GPa and superconducting. However, in thin films, YO is a semiconductor. Gd, Tb, and Ho monoxides were reported to be ferromagnetic semiconductors in thin films with critical temperatures higher than for EuO. Data for magnetic and thermodynamic properties on bulk rare earth monoxides are lacking, despite their importance in view of the potential application of these materials for spintronics and for thermodynamic assessments in multicomponent rare earth systems. The divalent state of Y and heavy lanthanides in monoxides, proposed in thin film studies, prompts further investigations on bulk samples.

## Figures and Tables

**Table 1:** Unit cell parameters for Th and thorium oxides.

Composition	a, Å	Notes	First Author, Year
Th fcc	5.084	fcc Th (*Fm*-3*m*) at RT	Hanawalt 1938 [79]
Th bcc	4.11(1)	bcc Th (*Im*-3*m*) at 1500 °C	Chiotti 1954 [80]
ThO	5.24	film on the surface of Th metal	Rundle 1947 [60]
ThO [Th bcc] *	4.31 *	(W)Th + ThO_2_ above ~1700 °C	Hoch 1954 [81]
ThO [ThO_2_] †	5.63(1) †	75 °C vacuum dry residual in HCl	Katzin 1958 [82]
ThO	5.302(3)	900 °C anneal of the residual in HCl	Ackerman 1973 [77]
ThO_0.75_	5.41	100 nm film on Th metal	He 2017 [28]
ThO_2_ flrt	5.5997	fluorite ThO_2_ (*Fm*-3*m*) at RT	Wyckoff 1963 [83]

* likely high-temperature Th bcc structure, see text for details. † likely dioxide, compare lattice parameter with fluorite ThO_2._

**Table 2:** Lattice parameters reported for rocksalt-type (*Fm*3*m*) rare earth monoxides (REO) synthesized in thin films (TF) and in bulk.

REO	*a*, Å (TF) *	Comment †	REO	*a*, Å *	Comment
LaO	5.22–5.31 §	~20 nm (0.98–1.02) [42]	LaO	5.144	golden, 4 GPa 900 °C [34]
CeO	5.15	~6 nm (1.01) [103]	CeO ‡	5.089	golden, 1.5 GPa 700 °C [96]
PrO	5.054	~10 nm (1.02) [104]	PrO	5.031	golden, 5 GPa 800 °C [96]
NdO	5.05–5.16 §	~20-40 nm (1.01–0.99) [40]	NdO	4.994	golden, 5 GPa 1000 °C [34]
				5.086	shock compression [102]
SmO	5.02	~70 nm (0.99) [43]	SmO	4.943	golden, 5 GPa 1000 °C [34]
EuO	5.152	~100 nm (MBE) [105]	EuO	5.144	dark red, 0 GPa [106]
	5.12	~100 nm Eu_0.9_La_0.1_O [105]		5.143	EuO_1.02_ 0 GPa [92]
GdO	5.00–5.02	~50 nm (0.99) [38]			
	5.03	~90 nm (1.00) Gd_0.90_La_0.10_O [38]			
TbO	4.97	~90 nm (0.99) [36]			
HoO	5.04	~20-90 nm (0.97) [37]			
YO	4.92–4.98 §	~90-200 nm (0.99) [44]			
YbO	4.87	~4-30 nm (0.99) [39]	YbO	4.877	1-6 GPa 600–1400 °C [95]
LuO	4.79	~150 nm (0.99) [41]			

* The lattice parameters are reported to the last significant digit. † The tetragonal distortion was induced by epitaxial growth (in plane lattice constant = *a*∙(k); the k values less than 1 correspond to compression, higher than 1 to tension); the films were grown by pulsed laser deposition (PLD), except EuO which was grown by molecular beam epitaxy (MBE). ‡ Synthesized by reaction of Ce with CeO_2_; Ce_2_O_3_ was also formed as a product. § The larger cell parameter may correspond to oxygen-deficient samples.

**Table 3:** Temperatures (in K) of magnetic ordering reported for rare earth monoxides (Curie temperatures Tc, and Néel temperatures (T_N_)) compared with RE metals and other rocksalt-type RE chalcogenides and pnictides.

RE	Metal *	REO †	RES ‡	RESe ‡	RETe ‡	REN ‡	REP ‡	REAs ‡	RESb ‡	REBi ‡
Pr	(25)	28								
Nd	(20)	19	8.2	10.6	10.2	27.6	11	10.6	15.5	25
Eu	(90)	79	(16.6)	(4.6)	(9.6)					
Gd	293	276	62	65	70	72	20	21	28	32
Tb	221(229)	231	45(49)	49(52)	51(63)	42(34)	9	12	14(16.5)	18
Ho	20(132)	131	17(21)		20	~15	5.5	4.8	5.5	5.7

* From Koehler (1965) [113], magnetic ordering in Eu from [114,115]. † Measured on epitaxial thin films from pulsed laser deposition (see Table 2 for references). ‡ From Vogt and Mattenberger (1993) [106,108].

## Data Availability

The data are available from contacted authors upon request.
